# LncRNA AFAP1-AS1 promotes growth and metastasis of cholangiocarcinoma cells

**DOI:** 10.18632/oncotarget.16880

**Published:** 2017-04-06

**Authors:** Xiuhui Shi, Hang Zhang, Min Wang, Xiaodong Xu, Yan Zhao, Ruizhi He, Min Zhang, Min Zhou, Xu Li, Feng Peng, Chengjian Shi, Ming Shen, Xin Wang, Xingjun Guo, Renyi Qin

**Affiliations:** ^1^ Department of Biliary-Pancreatic Surgery, Affiliated Tongji Hospital, Tongji Medical College, Huazhong University of Science and Technology, Wuhan, China

**Keywords:** long noncoding RNA, AFAP1-AS1, cholangiocarcinoma, proliferation, metastasis

## Abstract

We investigated the role of actin filament associated protein 1 antisense RNA1 (AFAP1-AS1) lncRNA in promoting cholangiocarcinoma (CCA). qRT-PCR analysis of patient samples showed that AFAP1-AS1 expression was higher in CCA tumors than matched adjacent non-tumor tissue. AFAP1-AS1 levels were also higher in CCA cell lines (HuCCT1 and TFK-1) than a normal biliary epithelium cell line (HIBEpic). AFAP1-AS1 knockdown in CCA cell lines using shAFAP1-AS1 reduced cell proliferation and colony formation in CCK-8 and colony formation assays, respectively. Cell cycle analysis demonstrated that AFAP1-AS1 knockdown resulted in G0/G1 cell cycle arrest and inhibition of S-G2/M transition compared to the controls. CCA cells transfected with shAFAP1-AS1 also exhibited reduced metastasis and invasiveness in Transwell and wound healing assays. This was further confirmed in xenograft experiments with nude mice using CCA cells transfected with shAFAP1-AS1 or control shRNA. AFAP1-AS1 knockdown cells produced smaller tumors, demonstrating that AFAP1-AS1 promotes tumor growth *in vivo*. AFAP1-AS1 knockdown also increased expression of actin filament associated protein 1 (AFAP1) and reduced cell stress filament integrity, as determined from western blot and immunofluorescence assays, respectively. These findings indicate that AFAP1-AS1 exerts oncogenic effects in CCA. We postulate that AFAP1-AS1 is a potentially useful diagnostic and prognostic biomarker and therapeutic target for CCA.

## INTRODUCTION

Cholangiocarcinoma (CCA) is one of the most aggressive and lethal tumors originating from malignant transformation of cholangiocytes and epithelial cells lining the intrahepatic and extrahepatic biliary ducts [1]. Nearly 80%–90% of CCAs are of extrahepatic origin that is further divided into perihilar (Klatskin tumor) and distal tumors based on their location within the extrahepatic biliary system [2]. CCA is an aggressive cancer with medial survival of less than 24 months after diagnosis. Curative surgery is recommended only for early-stage patients and is not available for advanced stage patients. Systemic chemotherapy with gemcitabine and cisplatin is standard practice for advanced stage patients. However, inspite of the combination chemotherapy, the 5-year survival rates in CCA patients remains less than 20-40% [3]. Therefore, there is greater need to identify novel therapeutic targets by deciphering the critical molecular mechanisms regulating CCA in order to improve patient survival times.

Long noncoding RNAs (lncRNAs), which are more than 200 nucleotides in length, are key regulators of cellular transcription [4]. A number of lncRNAs play pivotal roles in gene regulation during development and disease pathogenesis. Also, many lncRNAs have been recognized as oncogenes or tumor suppressors [5–7]. LncRNAs regulate gene expression either through transcriptional interference or by inhibiting the translation of RNA [8, 9]. The dysregulation of lncRNAs has been reported to play a vital role in diverse human diseases including cancers [10]. Recently, the mechanism and function of several lncRNAs such as HOTAIR, HULC, and MALAT1 were reported in hepatic carcinoma resulting in construction of lncRNA regulatory networks in hepatic carcinoma [11–13]. Similarly, we postulated existence of dysregulated lncRNAs that would promote CCA by regulating the key signaling pathways. Hence, identification of cancer-associated lncRNAs and their molecular and biological functions in CCA is important.

Actin filament associated protein1 antisense RNA1 (AFAP1-AS1) is a lncRNA derived from the antisense DNA strand in the AFAP1 gene locus, which regulates actin filament integrity and act as an adaptor protein linking Src family members and other signaling proteins associated with actin filaments [14, 15]. High AFAP1-AS1 levels were associated with malignancy, metastasis and poor prognosis of hepatocelluar carcinoma, pancreatic ductal adenocarcinoma, and gall bladder cancer [16–18]. Therefore, AFAP1-AS1 is a potential diagnostic and prognostic biomarker, as well as a therapeutic target in these cancers. However, the expression pattern and potential function roles of AFAP1-AS1 in CCA are not clear. In this study we investigated if AFAP1-AS1 was a novel prognostic indicator in CCA and explored the feasibility of lncRNA based diagnosis and gene therapy for this deadly disease.

## RESULTS

### AFAP1-AS1 is significantly up-regulated in CCA tissues and cell lines

**Figure 1:**
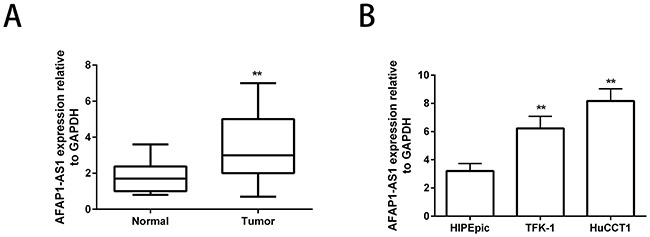
Enhanced AFAP1-AS1 expression in human CCA cell lines **(A)** Relative expression of lncRNA AFAP1-AS1 in matched CCA and adjacent non-tumor patient tissues. LncRNA AFAP1-AS1 expression was examined by qRT-PCR and normalized to GAPDH expression. **(B)** Expression levels of lncRNA AFAP1-AS1 in CCA cell lines, HuCCT1 and TFK-1, compared to HIBEpic. Data are represented as the mean ± SD from three independent experiments. **P<0.01. Student's t-test.

We examined the expression levels of AFAP1-AS1 in 20 CCA and adjacent normal patient tissues by qRT-PCR and observed that its expression was significantly upregulated in the CCA tissues (Figure 1A). Further, we compared the expression of AFAP1-AS1 in the CCA cell lines and a normal biliary epithelium cell line, HIBEpic by qRT-PCR and again observed that its expression was significantly higher in the CCA cell lines compared to HIBEpic (Figure 1B). These data demonstrated that the AFAP1-AS1 had an oncogenic role in CCA.

### Knockdown of AFAP1-AS1 inhibits CCA cell proliferation *in vitro*

**Figure 2:**
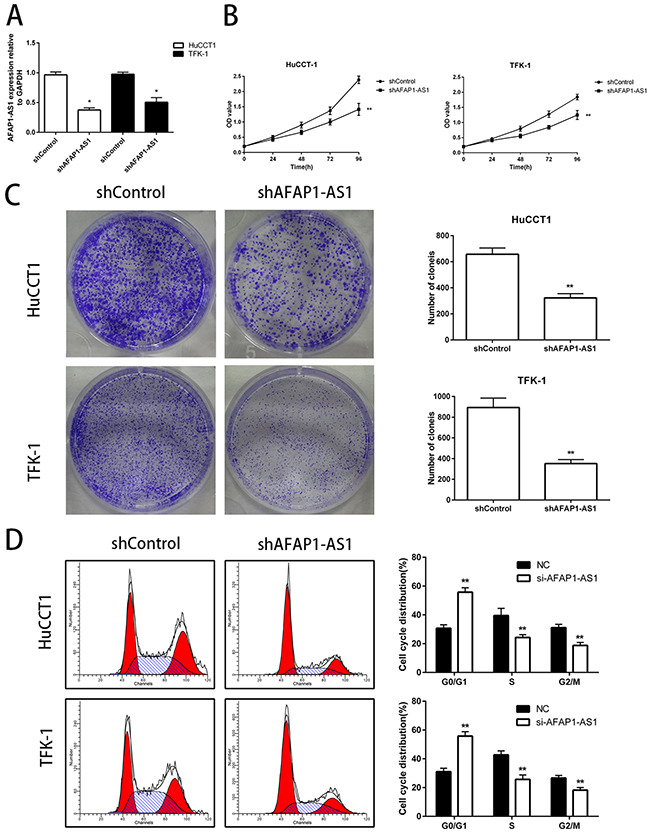
Knockdown of AFAP1-AS1 decreases *in vitro* proliferation of CCA cell lines **(A)** Expression of AFAP1-AS1 in HuCCT1 and TFK-1 cells infected with lentiviruses carrying either si-AFAP1-AS1 or control siRNAs were analyzed by qRT-PCR. The effects of knockdown of AFAP1-AS1 in cells on cell proliferation were examined by **(B)** CCK-8 assay and **(C)** colony formation assay. **(D)** Cell cycle profiles of propidium iodide stained HuCCT1 and TFK-1 cells transfected with NC and siAFAP1-AS1 siRNAs were analyzed by flow cytometry and the ratios of G0/G1, S and G2/M cell numbers were determined (left panel). The statistical results were shown on the right panel. Data are represented as the mean ± SD from three independent experiments. *P<0.05 and **P<0.01. Student's t-test.

To further examine the role of AFAP1-AS1 in CCA progression, we knocked down AFAP1-AS1 expression in HuCCT1 and TFK-1 cells by stable transfection of either shControl or shAFAP1-AS1 as shown in Figure 2A. Then, we performed cell proliferation assays using the CCK-8 assay kit and observed that knockdown of AFAP1-AS1 expression significantly suppressed cell growth in HuCCT1 and TFK-1 cells compared to the controls (Figure 2B). In addition, downregulation of AFAP1-AS1 significantly inhibited colony formation in HuCCT1 and TFK-1 cells (Figure 2C). Further, we assessed the effects of AFAP1-AS1 knockdown on the cell cycle of CCA cell lines by flow cytometry and observed that in comparison with the control, the AFAP1-AS1 knocked down cells showed an increase in the G0/G1 phase cells and a decrease in the S phase and G2/M phase cells (Figure 2D). This suggested that knock down of AFAP1-AS1 in the CCA cells resulted in G0/G1 cell cycle arrest and inhibition of S and G2/M cell cycle progression. Together, these data suggested that AFAP1-AS1 played a critical role in CCA cell proliferation *in vitro*.

### Knockdown of AFAP1-AS1 inhibits CCA cell migration and invasion *in vitro*

**Figure 3:**
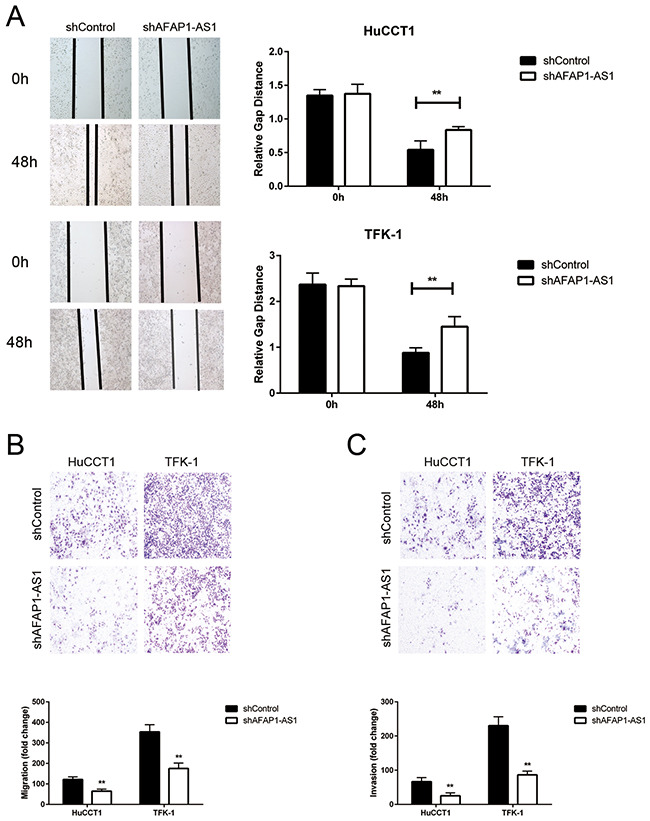
The effects of AFAP1-AS1 knockdown on migration and invasion of CCA cell lines **(A)** Representative images of wound healing assay show the migratory abilities of HuCCT1 and TFK-1 cells infected with lentivirus of shControl or shAFAP1-AS1 at 0h and 48h time points, respectively. **(B)** The Boyden chamber assay examining the migratory ability of HuCCT1 and TFK-1 cells infected with lentivirus of shControl or shAFAP1-AS1, respectively is shown. **(C)** The Boyden chamber assay examining the invasiveness of HuCCT1 and TFK-1 cells infected with lentivirus of shControl or shAFAP1-AS1, respectively is shown. Data are represented as the mean ± SD from three independent experiments. *P<0.05 and **P<0.01. Student's t-test.

The most important traits of cancers are unrestricted cancer cell growth and migration. Therefore, we evaluated the consequences of AFAP1-AS1 knock-down on CCA cell motility by wound healing and transwell assays. We observed that knockdown of AFAP1-AS1 markedly inhibited the migration of HuCCT1 and TFK-1 cells as visualized by wound healing assays (Figure 3A). Further, the transwell assay also showed that motility of CCA cell lines was significantly decreased in shAFAP1-AS1 transduced CCA cells compared to the control (Figure 3B, 3C).

### Inhibition of AFAP1-AS1 impaired CCA cell tumor growth *in vivo*

**Figure 4:**
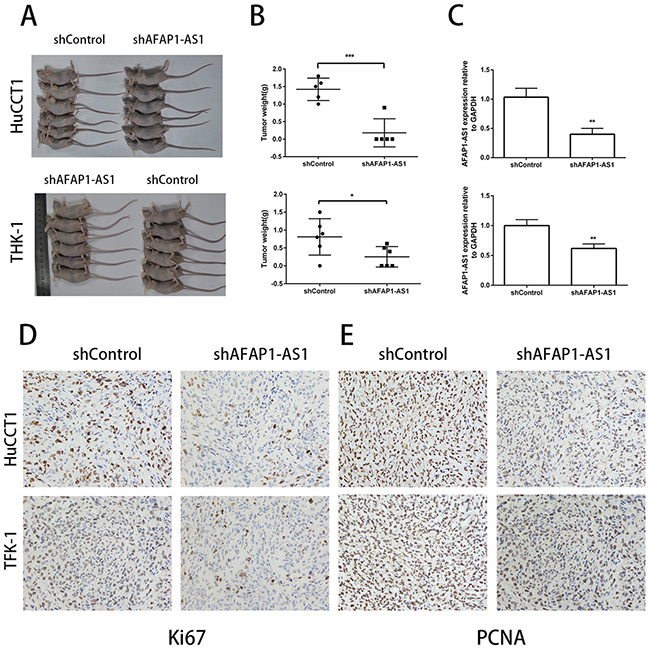
Inhibition of AFAP1-AS1 impairs tumorigencity of CCA cell lines *in vivo* **(A)** Images of tumors formed in nude mice were subcutaneously injected into the right axilla with cells infected with shControl lentiviral vector (containing scrambled control shRNA) or shAFAP1-AS1 lentiviral vector (containing shRNA targeting AFAP1-AS1). **(B)** The average tumor weights harvested from nude mice infected with shControl lentiviral vector or shAFAP1-AS1 lentiviral vector are shown. **(C)** The expression of AFAP1-AS1 in xenograft tumors harvested from nude mice infected with shControl lentiviral vector or shAFAP1-AS1 lentiviral vector as analyzed by qRT-PCR is shown. **(D, E)** Representative images of IHC staining showing expression of Ki67 and PCNA in xenograft tumor tissues from the shAFAP1-AS1 group or shControl group. *P<0.05, **P<0.01 and ***P < 0.001, Student's t-test.

Next, we determined the effects of AFAP1-AS1 on *in vivo* tumor growth by xenografting shControl or shAFAP1-AS1 transfected HuCCT1 and TFK-1 CCA cells into nude mice. We observed that both tumor volume (Figure 4A) and tumor weight (Figure 4B) were significantly decreased in mice xenografted with CCA cells with knocked down AFAP1-AS1. The average expression of AFAP1-AS1 in xenograft tumors was lower in the shAFAP1-AS1 group than the control group (Figure 4C). In addition, immunostaining data revealed that the tumors derived from the shAFAP1-AS1 group had significantly weaker Ki67 and PCNA expression than tumors derived from the control group (Figure 4D, 4E). These data further supported the role of AFAP1-AS1 in CCA cell growth *in vivo*.

### AFAP1-AS1 knockdown inhibited CCA metastasis in nude mice

**Figure 5:**
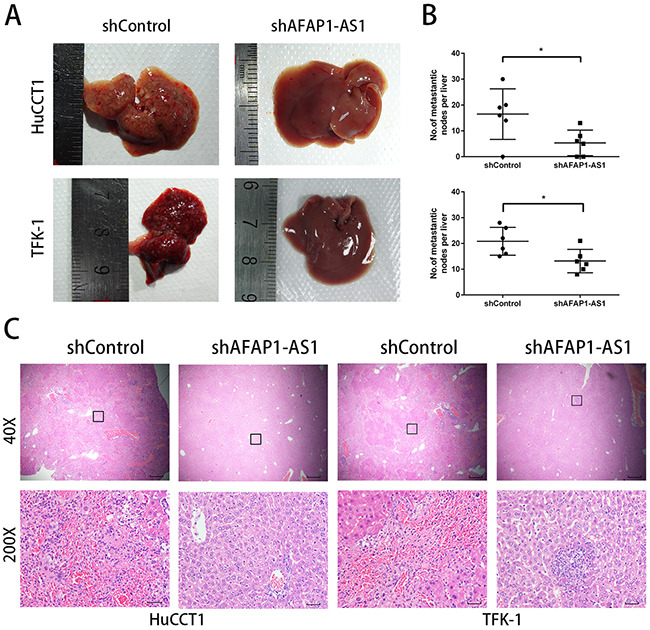
AFAP1-AS1 knockdown inhibits CCA metastasis in nude mice **(A)** Images from the liver metastasis model are shown. **(B)** Corresponding statistical analysis of average numbers of visible liver metastases are shown. The data are represented as mean ± SD (each data point represents a different mouse; n = 6 mice per group). **(C)** H&E-stained sections of liver metastasis tumor tissues. Rectangular boxes indicate clusters of micro metastatic cells in the liver. Images were acquired at 40X (Scale bar = 200um) and 200X (Scale bar = 50um).

To determine the effects of AFAP1-AS1 knockdown on metastasis *in vivo*, we inoculated HuCCT1 and TFK-1 cells transfected with shAFAP1-AS1 or shControl into the spleen of nude mice and assessed the number of metastasized tumor nodules in the liver. We observed that shAFAP1-AS1 significantly reduced the size and number of metastasized tumor foci (Figure 5A). Hematoxylin and eosin (H&E) staining of paraffin-embedded liver tissues also showed a decreased number and size of the metastatic foci in the mice inoculated with stable transfection mediated knockdown of AFAP1-AS1 (Figure 5B, 5C).

### AFAP1-AS1 knockdown induced the loss of cancer cell stress filament integrity

**Figure 6:**
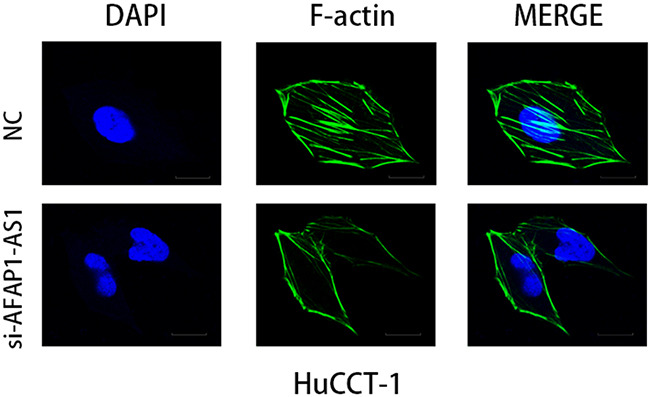
AFAP1-AS1 knockdown induced loss of stress filament integrity in CCA cell lines Immunofluorescence staining of HuCCT1 cells after transfected with NC and siAFAP1-AS1 siRNAs. Deficient stress fiber formation was observed in AFAP1-AS1 knockdown HuCCT1 cells is observed compared to controls 48h after cells were seeded on fibronectin coated glass coverslips. Images were acquired at 600X. Scale bar = 20μm.

Since AFAP1 is a modulator of actin filament integrity, we investigated the consequences of AFAP1-AS1 knockdown on actin filament integrity in HuCCT1 cells. No apparent changes were evident in morphology analysis by phase contrast microscopy of control and AFAP1-AS1 knocked down HuCCT1 cells (Figure 6). However, immunofluorescence analysis demonstrated that unlike the control HuCCT1 cells, the AFAP1-AS1 knockdown HuCCT1 cells demonstrated loss of stress filament integrity with F-actin not decorating the stress filaments (Figure 6).

### AFAP1-AS1 knockdown increased AFAP1 protein levels

**Figure 7:**
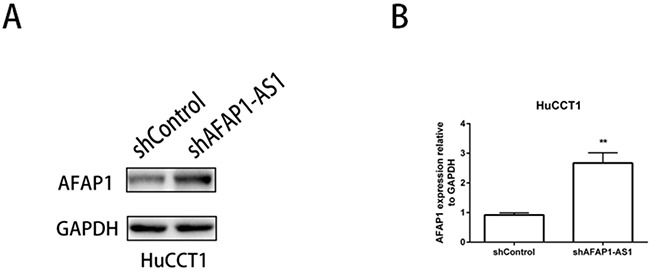
AFAP1-AS1 regulates the expression of AFAP1 protein and mRNA **(A)** Western blot analysis showing that AFAP1-AS1 knockdown upregulates AFAP1 protein levels in HuCCT1 cells. **(B)** AFAP1-AS1 knockdown increases AFAP1 mRNA levels in HuCCT1 cells as analyzed by qRT-PCR. Data are represented as the mean ± SD from three independent experiments. **P<0.01, Student's t-test.

Finally, we assessed the expression of AFAP1 protein and mRNA since AFAP1-AS1 is transcribed from the antisense strand of AFAP1 gene locus including an overlap between the second AFAP1-AS1 exon and the AFAP1 exons 14, 15 and 16. We observed that AFAP1 protein and mRNA expression levels were elevated when AFAP1-AS1 was knocked down in HuCCT1 cells (Figure 7A, 7B). This suggested that AFAP1-AS1 promoted cancer cell migration and invasion by interfering with AFAP1 expression.

## DISCUSSION

Despite a number of diagnostic advances and therapeutic strategies that have been achieved for CCA in recent years, overall survival rates of CCA patients is very poor due to recurrence and metastasis. Hence, novel diagnostic, prognostic and therapeutic approaches are essential to improve the survival rate of CCA patients and to provide further insights into the pathogenesis of this deadly disease [9].

Recent advances in genome-wide analyses have revealed that more than 97% of the total human genome are transcribed into short or long non-coding RNAs with limited or no protein-coding capacity [19]. Additionally, the roles of deregulated lncRNAs in human cancers have received considerable attention in the past few years [20]. Many studies have shown that lncRNAs are associated with many functional aspects of cell biology and there has been increasing attention to their role in tumorigenesis. For example, lncRNA GAS5 has been shown to be significantly down-regulated in hepatocellular carcinoma tissues [21]. lncRNA HOTAIR has been reported to be upregulated in breast cancer, pancreatic cancer, lung cancer, and gastric cancer, and high HOTAIR expression is associated with poor prognosis [22–24]. Previous studies demonstrated that the lncRNA AFAP1-AS1 was transcribed from the antisense DNA strand of the AFAP1 gene and regulated the invasion and metastasis of hepatocelluar carcinoma and pancreatic ductal adenocarcinoma cells [17, 18]. Recently, Hao et al. showed that AFAP1-AS1 mediated the malignant behavior of nasopharyngeal carcinoma cells by regulating the expression of several small GTPase family members and proteins that are part of the actin cytokeratin signaling pathway [25]. Recent studies also demonstrated that overexpression of AFAP1-AS1 predicted poor prognosis and promoted proliferation and invasion of gallbladder cancer cells [16]. However, the expression and functions of AFAP1-AS1 in CCA are unclear. Our study provides the first evidence that AFAP1-AS1 is significantly upregulated in CCA cell lines compared to normal HIBEpic cells. This further supported the finding that AFAP1-AS1 was up-regulated in the CCA tumors compared to adjacent normal patient tissues. Further, knockdown of AFAP1-AS1 resulted in decreased *in vitro* and *in vivo* proliferation and metastasis of CCA cancer cell lines, HuCCT1 and TFK-1, consistent with previous studies. These findings suggested that AFAP1-AS1 played an oncogenic role in CCA progression.

Previous studies have demonstrated that lncRNA activity was partly dependent on genomic location. LncRNAs like AFAP1-AS1 are oriented in an antisense direction to the protein-coding gene (AFAP1) in the opposite strand and regulated AFAP1 [26–28]. We demonstrated that AFAP1-AS1 expression increased AFAP1 protein levels and mRNA levels and confirmed this aspect. AFAP1 is an adapter molecule that links to other proteins such as SRC and PKC, thereby modulating changes to the actin filament integrity and inducing lamellipodia formation [14]. Therefore, the tumor metastatic effects of AFAP1-AS1 may be mediated by the altered AFAP1 protein levels.

In summary, our results showed that the lncRNA, AFAP1-AS1 was significantly upregulated in CCA tumors and cell lines and correlated with the *in vitro* and *in vivo* proliferation and metastasis. Our study demonstrates the potential of AFAP1-AS1 as a novel diagnostic and prognostic indicator and a therapeutic target for CCA. Further studies are necessary to unravel the detailed mechanism and function of AFAP1-AS1 in CCA in order to pursue an lncRNA-targeted therapeutic strategy for this deadly disease.

## MATERIALS AND METHODS

### Patients and samples

CCA tissues and matched adjacent normal bile duct tissues were obtained from 20 patients that underwent surgery between Jan 2010 and Nov 2016 in Tongji Hospital, Wuhan, China. All samples were processed by two professional pathologists. The fresh tissue specimens were snap frozen in liquid nitrogen and then stored at -80°C prior to RNA isolation. There was no pre-operative treatment prior to surgery. All patients signed the informed consent before surgery. This study was approved by the Human Ethics Committee of Tongji Hospital at Huazhong University of Science and Technology University (Wuhan, China) and carried out in accordance with the Declaration of Helsinki.

### Cell lines and cultures

The human cholangiocacinoma cell lines (HuCCT1 and TFK-1) and normal biliary epithelium cell line HIBEpic were grown in RPMI 1640 (Gibico, Carlsbad, CA, USA) medium supplemented with 10% fetal bovine serum (Gibico, Carlsbad, CA, USA) and 1% penicillin/streptomycin ((Beijing Solarbio Science & Technology Co., Beijing, China) at 37 °C and 5% CO2.

### Knock down of AFAP1-AS1 by shRNA transfection

For the knockdown experiments, siRNA targeting AFAP1-AS1 RNA (5’-AACACCAATCCCAAGAGGT GA-3’) and siRNA control (5’-TTCTCCGAACGTGT CACGT-3’) duplexes were purchased from the RiboBio, Guangzhou, China. Recombinant lentiviruses containing AFAP1-AS1(GenBank access number: NR_026892.1) expressing AFAP1-AS1-shRNA and AFAP1-AS1-specific cDNA were purchased from Genechem Co., Ltd (Shanghai, China). The GV248 vector (hU6MCS-Ubiquitin-EGFP-IRES-puromycin) used for the stable expression of shRNA against AFAP1-AS1 and a fluorescent marker (GFP-RFP fusion protein) contained a puromycin resistance gene. The negative control (NC) sequence was indicated as “NC” and had no homology to any human genomic sequences. Lenti-viral transfection was conducted according to the GenePharma Recombinant Lentivirus Operation Manual (http://www.genepharma.com). HuCCT1 and TFK-1 cells (1 × 105 cells/well) were seeded into 6-well plates for 24 hours, and after the addition of polybrene (8 μg/ml), the cells were infected with 2 μl of concentrated lentivirus for 72 hours. Cells were selected for 2 weeks with the addition of puromycin (5μg/ml, Sigma-Aldrich, St, Louis, USA) to generate stablem-onoclonal cell lines. Cells were selected for 2 weeks by the addition of puromycin to generate stable monoclonal cell lines. The expression of AFAP1-AS1 was confirmed by qRT-PCR.

### Quantitative real-time PCR (QRT-PCR) analysis

Total RNA was extracted from CCA cells using a TRIzol kit (Invitrogen, Carlsbad, CA, USA) according to the manufacturer's instructions. Complementary DNA (cDNA) was synthesized using 2 μg of the total RNA according to the instructions of the reverse transcriptase kit (Takara Bio, Inc., Dalian, China) in a LifePro Thermal Cycler (Hangzhou Bioer Technology Co. Ltd., Hangzhou, China). Then, cDNA samples (2 μl) were subjected to qRT-PCR using a SYBR®Premix EX Taq kit (Takara Bio, Inc., Dalian, China) for 40 cycles in a CFX ConnectTM Real-Time System (Bio-Rad, Hercules, CA, USA). GAPDH wasused as the internal control. The forward and reverse primers for AFAP1-AS1 were 5′-ATGGGGTAACTCAAAAAGCCTG-3′ and 5′-GCAGCAATTCAGAGCCAGTC-3′, respectively; the forward and reverse primers for AFAP1 were 5′-AGAGTGTCCTCCTCCACCAA-3′ and 5′-CTTGG CCTCTGATTTGGAAC-3′, respectively;the primers for GAPDH were 5′-GGAGCGAGATCCCTCCAA AAT-3′ and 5′-GGCTGTTGTCATACTTCTCATGG-3′, respectively. The cycle threshold (Ct) of different genes was first normalized to GAPDH for the same sample, and fold changes were calculated through relative quantification (2-ΔΔCt).

### Cell proliferation assay

A CCK-8 kit (Dojindo Laboratories Co. Ltd, Kumamoto, Japan) was used as a colorimetric assay to assess cell viability. Briefly, cells (5 × 10^3^ cells/well) were seeded into 96-well plates with 100 μl per well of RPMI1640 culture medium supplemented with 10% FBS and the indicated reagents. Each sample had six replicates. At the indicated time points, the medium was replaced by 100 μl fresh culture medium, and 10 μl CCK-8 solution was added to each well. Plates were incubated for 1-4 hours at 37°C before the absorbance was recorded at 450 nm using a Quant ELISA Reader (BioTek Instruments, USA). The percentage of viable cells was calculated according to the following method in a previously published report [29]: Survival Rate % =(ODtreated − ODblank)/(ODcontrol − ODblank) × 100%. The IC50 was calculated using probit regression analysis. The tests were repeated at least 3 times.

### Colony formation assay

Cells (500 cells/well) were seeded into 6-well plates and cultured for 2 weeks. After fixation in 4% paraformaldehyde for 10 minutes, cells were stained with 1% crystal violet. Colonies with diameters greater than 100 μm were counted, and experiments were run independently in triplicate.

### Wound healing assay

Cells were seeded into 6-well plates and cultured until they reached sufficient confluence. The cell monolayers were scratched manually with a 200 μl pipette tip. The plates were washed with PBS twice to remove floating cells. Cells were then incubated in RPMI-1640 supplemented with 1% FBS for 48 hours after the scratches were generated. Images of 6 random fields were captured by phase contrast microscopy (Niko Corporation) for quantitative analysis. The area into which the cells migrated was measured using the Image Pro Plus v6.0 software package (Media Cybernetics Inc., Bethesda, MD, USA).

### Matrigel invasion assay

Tumor cell invasion capacity was assessed using Transwell Cell Culture Inserts (8μm pore size, BD Biosciences, New Jersey, USA) in 24 well plates. A total of 1×10^5^ cells in 100μl of serum-free medium were added to the top chamber. The bottom well contained RPMI-1640 with 20% FBS. The cells were further incubated for 48h at 37°C. Then, the cells that invaded through the filter pores were fixed with methanol, stained with hematoxylin and observed under a microscope. The average number of invasive tumor cells were counted from 5 randomly selected 20x fields for each experiment.

### Cell cycle detection

For cell cycle analysis, Cells (5 × 103 cells/well) were seeded into 6-well plates and allowed to adhere overnigh and than, transfected with NC and siAFAP1-AS1 siRNAs. After 48 hours, cells were harvested and fixed in 75% ethanol at -20°C for 24h. After being washed with PBS, the cells were incubated at 37°C with RNaseA for 30 min followed by propidium iodide (PI, BD, USA) and incubated at room temperature in the dark for 30 min. Cell cycle was analyzed by a FACScan flow cytometer (Biosciences, San Jose, CA, USA) and ModFit 3.0 software (Verity Software House, Topsham, ME, USA).

### Western blotting

WB analysis was performed as previous described [30]. The following antibodies were used to determine AFAP1 content in cells: Rabbit monoclonal AFAP1 (cat. 21093-1-AP, Proteintech, Wuhan, China), Mouse mAb for GAPDH (cat. 10494-1-AP, Proteintech, Wuhan, China), goat anti-rabbit and rabbit anti-mouse secondary antibodies were purchased from Boster (Wuhan, China). The intensity of the protein band was densitometrically quantified using Image J software (version 1.50i). The ratio of the intensity of AFAP1 and the control GAPDH was used to determine relative AFAP1 levels in each cell type analyzed.

### Immunofluorescence

Cells were fixed in 4% paraformaldehyde for 20 min, permeabilized with 0.5% Triton X-100 for 5 min and blocked in phosphate-buffered saline (PBS) containing 5% fetal bovine serum for 30 min. Then, the cells were incubated for 1h with phalloidin-FITC (Molecular Probes, Beyotime, China) followed by three washes with PBS and then stained with 4’, 6-diamidino-2-phenylindole (DAPI) for 10 mins at room temperature. Immunofluorescence images were collected with a confocal fluorescence microscope (UltraView Vox; PerkinElmer, Waltham, MA, USA).

### Animal experiments

All animal experiments were approved by the Committee on the Ethics of Animal Experiments of HUST (Permit no. 2016-S014). All animal experimental procedures were in accordance with the Guide for NIH and the institutional ethical guidelines for animal experiments. All surgical procedures were conducted under sodium pentobarbital anesthesia and minimal animal suffering. For the tumorigenicity assay, TFK-1 and HuCCT1 cells (2 × 10^6^ cells, suspended in 100 μl RPMI-1640 without FBS) were subcutaneously injected into the upper right flank of nude mice (4-6 week-old, BALB/c/nu, female). Tumor sizes were measured by a vernier caliper every three days. For the metastasis assay, TFK-1 and HuCCT1 cells (2 × 10^6^ cells, suspended in 100 μl RPMI-1640 without FBS) were injected into the spleen of nude mice. All of the *in vivo* experiments were performed in specific pathogen-free (SPF) conditions. After 6 weeks, mice were sacrificed 6 weeks after inoculation and the tumor tissues were harvested, weighed, imaged, embedded in 10% paraffin, and subjected to IHC staining.

### Statistical analyses

The results of continuous variables are presented as mean ± SD unless otherwise stated. Treatment groups were compared using independent sample t-tests. Pairwise multiple comparisons were performed by one-way ANOVA (two-sided). P < 0.05 was considered statistically significant. All analyses were performed using IBM SPSS Statistics software version 17.0 (Chicago, IL, USA).
