# Correction: LncRNA AFAP1-AS1 promotes growth and metastasis of cholangiocarcinoma cells

**DOI:** 10.18632/oncotarget.28216

**Published:** 2022-05-19

**Authors:** Xiuhui Shi, Hang Zhang, Min Wang, Xiaodong Xu, Yan Zhao, Ruizhi He, Min Zhang, Min Zhou, Xu Li, Feng Peng, Chengjian Shi, Ming Shen, Xin Wang, Xingjun Guo, Renyi Qin

**Affiliations:** ^1^Department of Biliary-Pancreatic Surgery, Affiliated Tongji Hospital, Tongji Medical College, Huazhong University of Science and Technology, Wuhan, China; ^*^These authors have contributed equally to this study

**This article has been corrected:** In Figure 4D, the image of TFK-1 in the ‘shControl’ column contains an accidental overlap of the image of TFK-1 in the ‘shAFAP1-AS1’ column of Figure 4E. The corrected Figure 4, produced using the original data, is shown below. The authors declare that these corrections do not change the results or conclusions of this paper.

Original article: Oncotarget. 2017; 8:58394–58404. 58394-58404. https://doi.org/10.18632/oncotarget.16880

**Figure 4:**
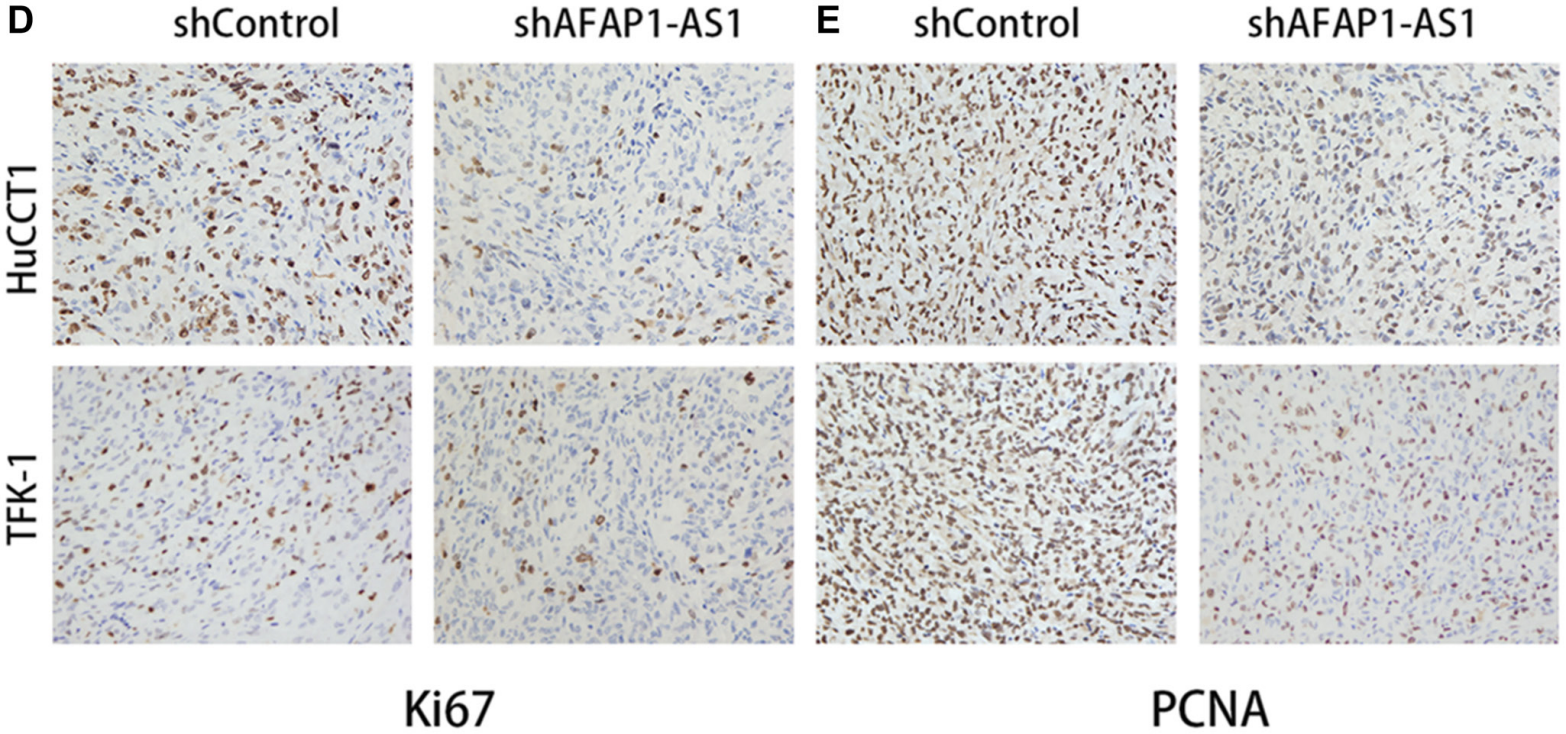
Inhibition of AFAP1-AS1 impairs tumorigencity of CCA cell lines *in vivo*. (**D**, **E**) Representative images of IHC staining showing expression of Ki67 and PCNA in xenograft tumor tissues from the shAFAP1-AS1 group or shControl group. ^*^ *P* < 0.05, ^**^ *P* < 0.01 and ^***^ *P* < 0.001, Student’s *t*-test.

